# Vegetation height estimation using ubiquitous foot-based wearable platform

**DOI:** 10.1007/s10661-020-08712-5

**Published:** 2020-11-21

**Authors:** Sofeem Nasim, Mourad Oussalah, Bjorn Klöve, Ali Torabi Haghighi

**Affiliations:** 1 Centre of Machine Vision and Signal Processing, Faculty of Information Technology, University of Oulu, Oulu, Finland; 2 Water, Energy and Environmental Engineering Research Unit, University of Oulu, Oulu, Finland

**Keywords:** Vegetation height, Machine learning, Ubiquitous sensor platform

## Abstract

Vegetation height plays a key role in many environmental applications such as landscape characterization, conservation planning and disaster management, and biodiversity assessment and monitoring. Traditionally, in situ measurements and airborne Light Detection and Ranging (LiDAR) sensors are among the commonly employed methods for vegetation height estimation. However, such methods are known for their high incurred labor, time, and infrastructure cost. The emergence of wearable technology offers a promising alternative, especially in rural environments and underdeveloped countries. A method for a locally designed data acquisition ubiquitous wearable platform has been put forward and implemented. Next, a regression model to learn vegetation height on the basis of attributes associated with a pressure sensor has been developed and tested. The proposed method has been tested in Oulu region. The results have proven particularly effective in a region where the land has a forestry structure. The linear regression model yields (*r*^2^ = 0.81 and RSME = 16.73 cm), while the use of a multi-regression model yields (*r*^2^ = 0.82 and RSME = 15.73 cm). The developed approach indicates a promising alternative in vegetation height estimation where in situ measurement, LiDAR data, or wireless sensor network is either not available or not affordable, thus facilitating and reducing the cost of ecological monitoring and environmental sustainability planning tasks.

## Introduction

Vegetation height is a key indicator for many terrestrial ecosystems linked to habitats, their biodiversity, and biomass structure (Hyde et al. 2006; Dong and Wu 2008; Nilsson 1996). Indeed, vegetation height is considered one of the most important forest properties and a fundamental characteristic for several areas of ecological studies; particularly, fire modeling, biodiversity monitoring, and disaster management where it can be utilized for classification of land cover or estimating forest age and habitat quality. For instance, vegetation height is highly correlated with vegetation biomass (Hyde et al. 2006), which is the fundamental element of the carbon cycle and a substitute for fuel loading estimation (Finney 1998).

Short vegetation, known as herbaceous vegetation, plays a key role in determining the confined livestock grazing and climatic variability as agents of vegetation change (Fuhlendorf et al. 2001). Conventionally, herbaceous vegetation height is measured using handheld devices such as hypsometers (for mature trees) or measuring poles (for seedlings and low vegetation) through field campaigns (Payero et al. 2004; Weltz et al. 1994). However, these methods are time consuming while incurring high labor cost, which, in turn, limit their ability to perform mapping at fine scales. Among popular alternatives to these approaches, one shall mention the imaging and radar-based methodologies. LiDAR, referred to as a 3D laser scanner, is recognized to be one of the most efficient alternate for recording vegetation data through airborne sensors (Nilsson 1996; Kwak et al. 2007; Lefsky et al. 2005), while LiDar provides highly efficient measurements at a footprint level of observation for forest structure. Nilsson (1996) estimated vegetation height on mountainous region of China, by calculating various vegetation indices using the Landsat data in collaboration with the LiDAR satellite data. Stojanova et al. (2010) estimated the vegetation height in a Slovenian forest region using Landsat imagery segment measurement, together with LiDAR data. Yanhong et al. (2010) used the vegetation indices obtained from the Landsat satellite data to provide an approximation of the vegetation height in an inland river basin of China. A comparative study conducted by Hyde et al. (2006) using airborne LiDAR, SAR/InSAR, satellite Landsat ETM+, and Quickbird examined the estimation of canopy height in a forest structure in the USA. The results indicated that LiDAR provides a better accuracy in height estimation as compared to other single sensor-based estimation. Besides, combining the LiDAR data with Landsat yielded more enriched results. Likewise, Wang et al. (2011) employed a MODIS sensor with a moderate resolution imaging spectroradiometer for estimating vegetation height in a large forest region area of the USA and Costa Rica. Nevertheless, such techniques are alleged to be less effective and challenging with the short vegetation height mainly because short vegetation height does not provide detectable increase among the initial and last LiDar return (Petzold et al. 1999). This is also reinforced by Rosso’s study (Rosso et al. 2006) which compared measurable errors of dataset obtained from LiDar and ground measurement in order to characterize wetland topology, and concluded that LiDar-based analysis has no potential to influence the ground underneath vegetation. Nevertheless, although LiDar- and satellite-based techniques are reliable, they only provide spatial coverage at a large resolution while requiring increasing demand of operational costs and labor, which calls for future research on the issue.

In this respect, our study aims to overcome the challenges of such operational costs experienced in remote sensing technologies and wireless sensor network (WSN) deployment infrastructure, by utilizing low-cost sensors in the form of wearable devices. The goal is to introduce a new perspective in data acquisition and analysis from a low-cost multisensory handed device through a wearable platform for estimating the vegetation height. For this purpose, we propose a novel foot-based wearable platform that records measurements like plantar pressure, humidity, and inner temperature. The intuition behind the proposal is similar in spirit to touch-sensing technology, which allows us to recognize objects by solely touching the various parts of the object(s). Similarly, we hypothesize that soil properties, including vegetation height, can also be approached by exploring the foot-sensing modalities as measured by some foot-wearable platform. For this goal, a simple regression model was developed in order to carry out the estimation process. An experimentation is performed in the Oulu region in an area involving various vegetation types and heights. Comparison with traditional methods is also carried out for illustration purpose. Especially, the pressure sensors are found to positively correlate with the vegetation height measured using a handheld device.

In this respect, extending our earlier findings at Nasim et al. (2019), this paper demonstrates the feasibility of an affordable foot-based ubiquitous platform for vegetation height estimation, hinting the development of new technology for soil analysis and remote environment monitoring. The second section of this paper highlights the Section “Study area”. The detailed Section “Methodology” is described in the third section. Section “Results and discussion” is reported in the fourth section, and finally, Section “Conclusion” is drawn in the last section.

## Study area

**Fig. 1:**
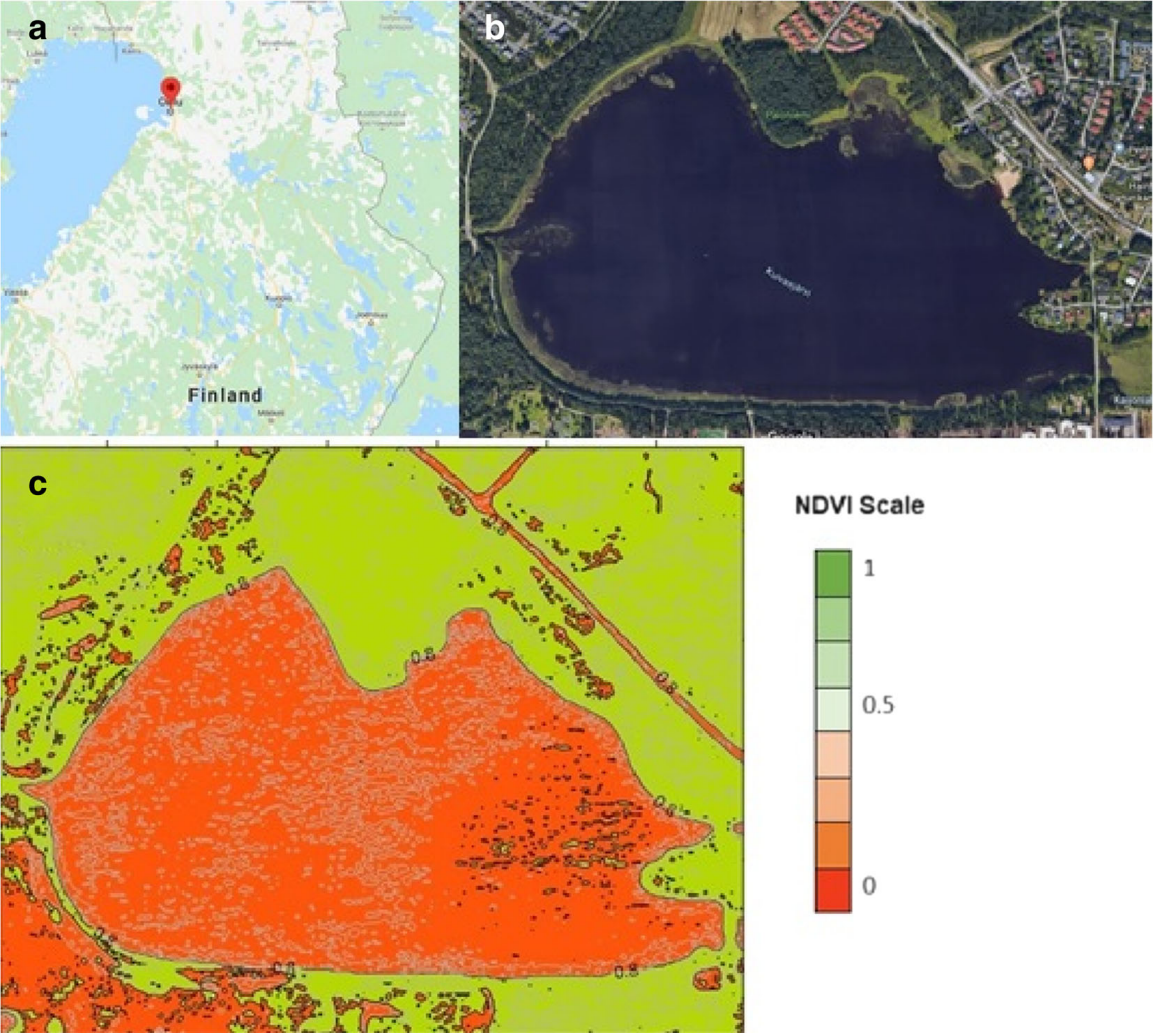
Study area where the experiment was conducted. **a** Finland’smap; **b** satellite view of Kuivasjärvi; **c** NDVI map of the study area (July 2018)

**Fig. 2:**
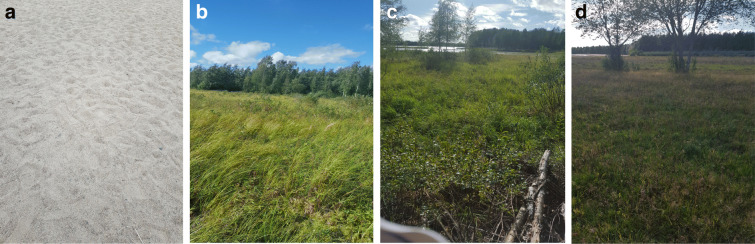
Study areas near Kuivasjärvi, Oulu, Finland: **a** site 1; **b** site 2; **c** site 3; **d** site 4

This study has been conducted in an 8-type soil variety area in Oulu region, Finland (Fig. 1) which highlights distinct vegetation height levels. Typically, Normalized Difference Vegetation Index (NDVI) is a standard way to measure healthy vegetation. High (low) NDVI values indicate healthy (poor) vegetation quality. Besides, the existence of several repositories and open data where NDVI values are publicly available provides us with an efficient tool to guide the selection of the study area in a way to ensure useful differentiation. Accordingly, we have selected the study area based on NDVI index and the Google Earth location to ensure the variability in vegetation height at each site. However, the amount of variation is quite difficult to estimate solely using the NDVI index. See Fig. 1 for an overview of the NDVI map around the study region together with Google Maps view of the area (Fig. 2). The types of vegetation encountered in the study area are summarized in Tables 1 and 2.

**Table 1:** Detail of study area locations including its vegetation types and 5 structures

Study area	X coordinates	Y coordinates	Vegetation type	Structure presents
Site 1	65.069220	25.483756	Sand	None
Site 2	65.070162	25.480847	Grassland	A, B, C
Site 3	65.071213	25.478959	Forest	A, B, C
Site 4	65.072667	25.471560	Grassland	A, C
Site 5	65.071420	25.465581	Forest	A, B
Site 6	65.063355	25.475950	Forest	A, B, C
Site 7	65.064639	25.472442	Forest	A, B, C
Site 8	65.064445	25.467862	Road	None

**Table 2:** Vegetation structures present in study area

Structure type	Description
A	Mixture of woody and herbaceous plants
B	Woody plants dominate herbaceous plants
C	Herbaceous plants

**Fig. 3:**
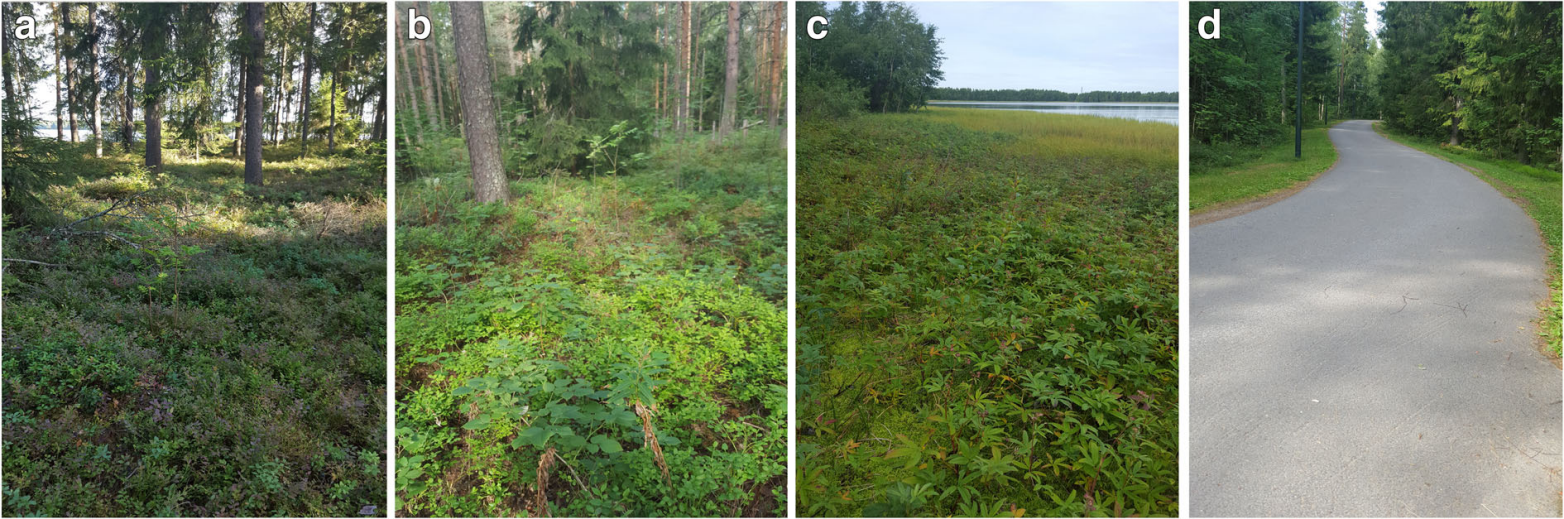
Study areas near Kuivasjärvi,Oulu, Finland: **e** site 5; **f** site 6; **g** site 7; **h** site 8

It has been noted that almost all of the study area is filled with an assortment of different structures such as a mixture of woody and herbaceous, and herbaceous plants. On the other hand, woody plants are found to dominate herbaceous plants in general, while important concentration of herbaceous vegetation is observed at each study area (Fig. 3).

## Methodology

### Overall system design

**Fig. 4:**
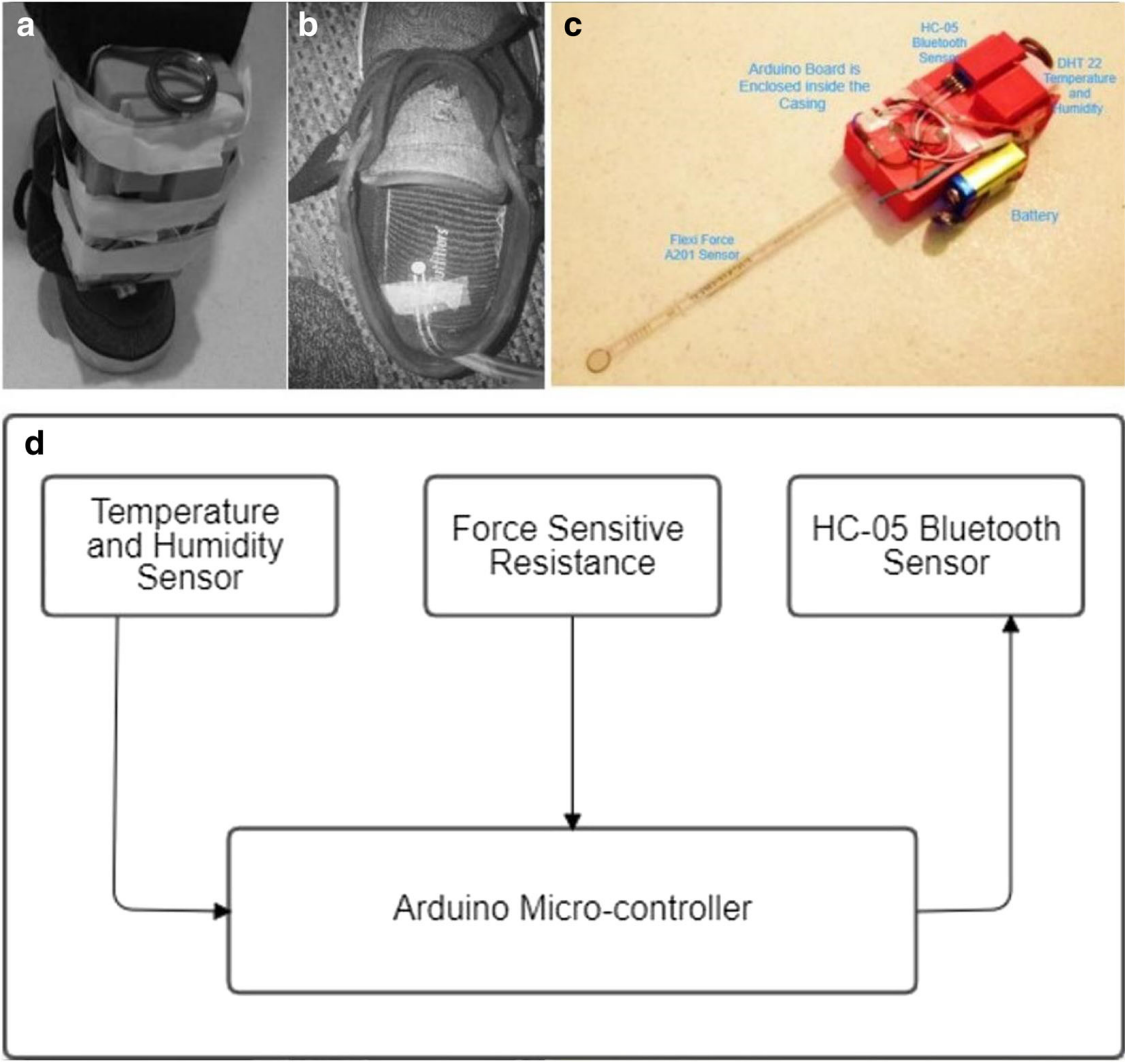
**a** Wearable-foot platform attached to user’s foot. **b** Pressure sensor placement. **c** Image view of the developed system. **d** Block diagram representation

The overall wearable platform highlighted in Fig. 4c consists of three sensors and one micro-controller. It includes temperature, humidity, and pressure sensors together with a Bluetooth wireless sensor. In general, temperature and humidity sensors are commonly employed in monitoring environmental conditions, which provide insights to comprehend soil properties. The use of pressure sensor is rather new in remote monitoring applications, although it is widely employed in other applications, e.g., health-related applications. Intuitively, one expects that different pressure signal patterns generated from the force sensor when subjected to different types of canopy cover provides us with useful insights about soil and vegetation patterns as well. In this study, the canopy cover refers to the proportion of the study site covered by a specific vegetation type. The Arduino micro-controller retrieves the state and outputs of various sensor signals, processes the decision-signal output, and transmits it to a mobile platform through a serial port.

The weight of the developed platform is only around 110 g, which makes it not exceed that of a normal winter or sport shoes. Therefore, this does not affect the movement of the user and his normal walking patterns should not be affected.

The synergy of the three sensors, namely, flexi-force sensitive resistor, temperature and humidity, and Bluetooth, is utilized for strengthening the overall building of the wearable platform (Fig. 4d). The Bluetooth sensor is employed solely for transmission purpose. An Android application is also developed to store the readings obtained from the sensors on a mobile application

and perform advanced calculus on a cloud platform. As a working principle of the system, DHT22 collects the information of temperature and humidity from the external surroundings and transmits the output as a digital signal via MaxDetect 1-wire to the Arduino board. The Arduino receives 40 bits in the form of a digital signal where the first 16 data bits represent Relative Humidity. The next 16 data bits represent the Temperature, and, finally, the last 8 bits correspond to Check Sum bits.

Whereas, the force is calculated, when it is applied on the sensing area of the Force Sensor Resistor, which in turns, creates a change in the analogue output voltage. The calculation of the output voltage is given by:

1 $$ \begin{array}{@{}rcl@{}} Vo = Vs\ast R/(R+Fsr) \end{array} $$

**Fig. 5:**
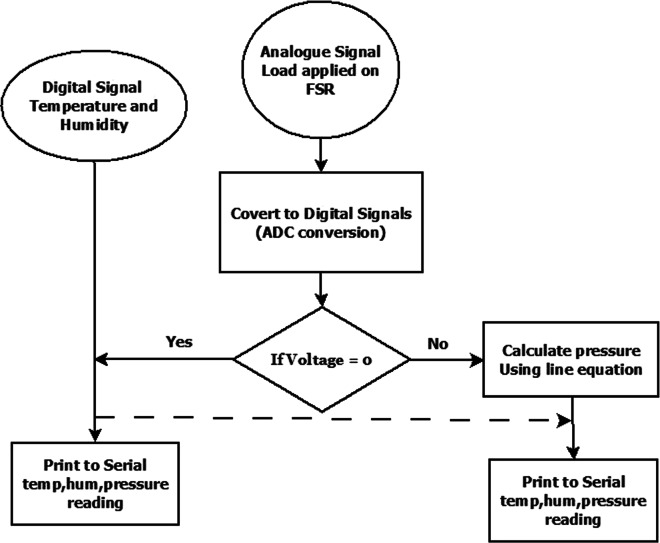
Flowchart of the pressure-sensory system

That is, the voltage is proportional to the inverse of the FSR resistance. More specifically, at first, the analogue voltage is transformed to a digital signal using ADC conversion function provided by the Arduino library. Then, the obtained digital voltage is converted into a force using the best-fit linear Eq. 2, derived during the calibration process. The complete detail of acquiring pressure, temperature, and humidity measure is described in the flowchart (Fig. 5). Data logging part takes place after the data has been processed.

**Fig. 6:**
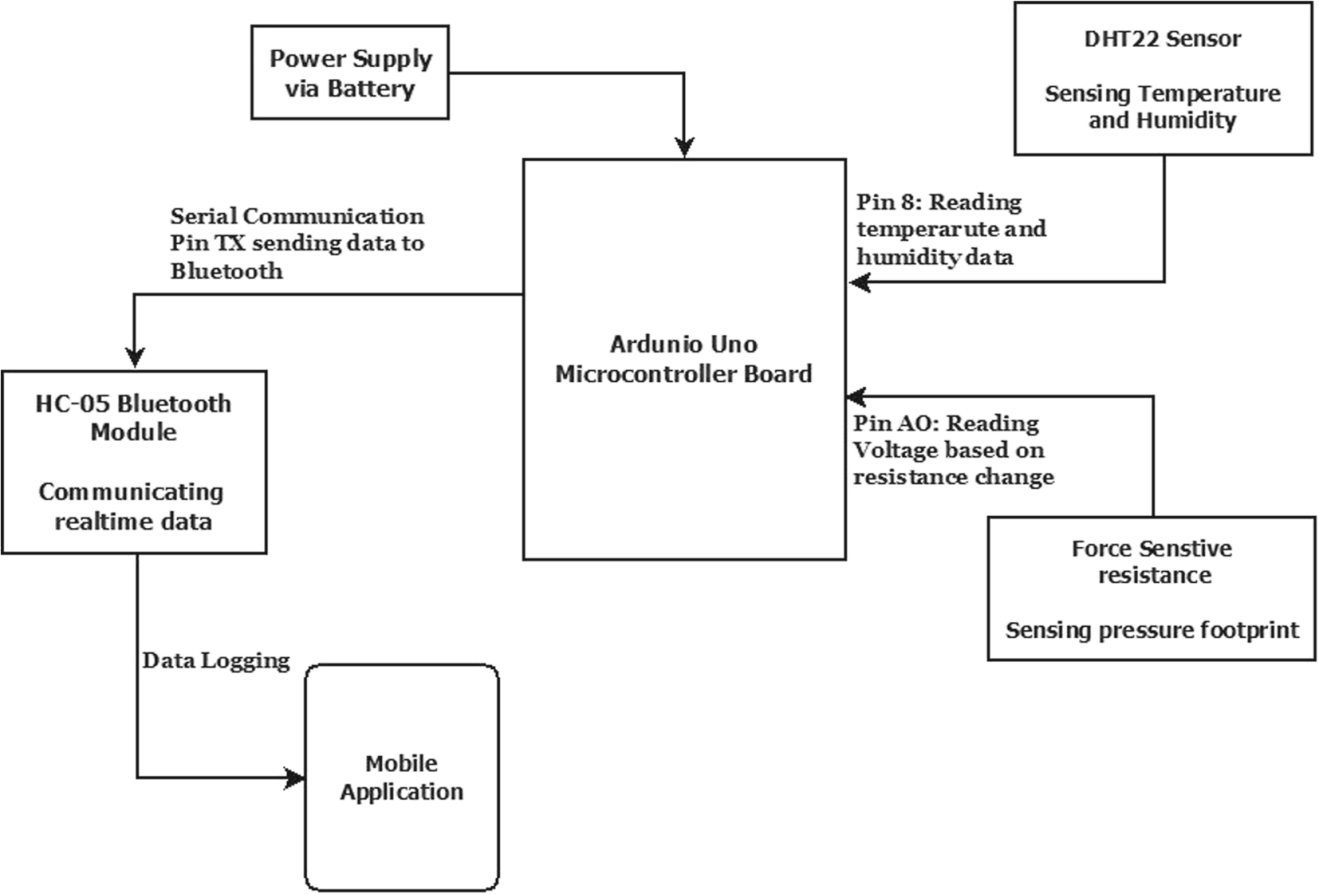
Block diagram of data transmission

The Arduino board transmits the data of time interval, temperature, humidity, force, and voltage through the serial port, where the Bluetooth connected to TX pin receives the output and wirelessly transmits it to the mobile application. The application stores it as a text file, which is then converted to an Excel file in a PC for calculating the results. The block diagram shown in Fig. 6 details the various data communication modules.

### Arduino microcontroller

Arduino platform is a widely used open-source electronic prototyping platform with a single board microcontroller, which is flexible and user-friendly in term of hardware and software components. Arduino UNO board is based on ATmega328, with 14 digital input/output pins, 6 analogue pins, and 16 MHz of clock speed, which provides the ample setup for various sensors, connection, and transmission.

### Sensors

The choice of the sensors (temperature, humidity, flexi-force, and Bluetooth) embedded in our platform has been mainly motivated by low cost, compatibility with Arduino module, and good cost/quality balance. Figure 8 provides a circuit connection to Arduino module of each of the three sensors, respectively. The detail of individual sensor is reported in the next subsections.

#### Temperature and humidity

We have chosen DHT 22 temperature and humidity sensor (Fig. 8a) due to its high reliability, good stability, and compatibility with Arduino platform. The DHT22 sensor consists of two parts: a capacitive humidity sensor, which is responsible for measuring the humidity, and a thermistor that measures the temperature of its surroundings. The sensor has the capacity to measure the temperature in the ranges from − 40 to + 125 °C with ± 0.5° accuracy, offering excellent quality, fast response, anti-interference ability, and cost-effectiveness. The DHT 22 temperature sensor comprises three pin VCC, GND, and data pin. It can be easily interfaced with Arduino board via connecting the VCC pin to 5v, GND to GND pin, and data pin to any of the digital pin of the Arduino board. On the software part, the DHT library is available in Arduino website. This enables us to read the temperature from the sensor and display it in the serial monitor.

#### Flexi-force sensor (pressure sensor)

Flexi-force sensor (Fig. 8b), also referred to as the force sensitive resistor, is used for calculating the pressure value. It operates on changing its resistance when an external force, pressure, or stress is applied. Tekscan flexi-force A 201 became nowadays quite a standard and among most popular instruments for measuring force in wearable platforms. A fixed value resistor of 1 Mohm is connected in a series with the FSR resistance. The connection of FSR with Arduino is established by joining one end to the power pin and the other end to the fixed value resistor ground, the point where the resistor is connected to analogue pin of Arduino board (see Fig. 8e).

**Fig. 7:**
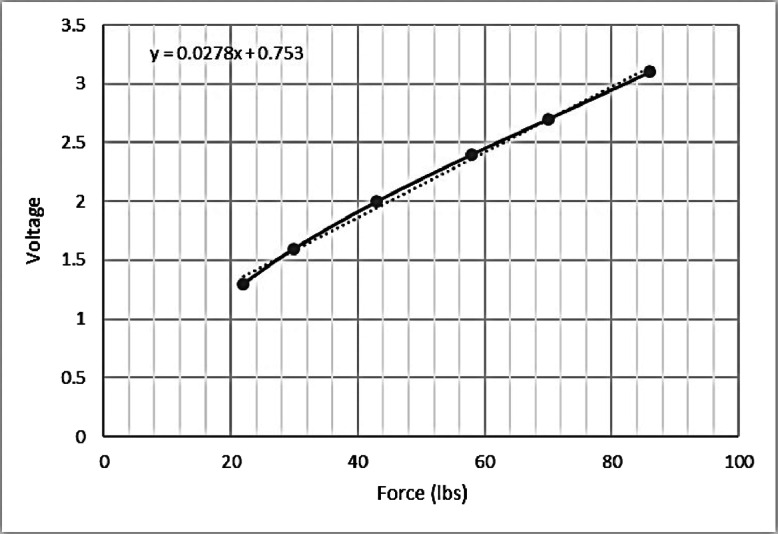
Linear interpolation of measurements for value estimation of forces

In order to determine the force of unknown loads, the equation for the best fit is to be derived. For this purpose, a set of input–output voltage measurements should be carried out. Next, voltage–force graph is plotted, as in Fig. 7, and the best linear fit is identified. Typically, in order to filter out potential outliers and increase consistency of measurements, in agreement with manufacture recommendation, a voltage vs. force graph is plotted in order to find the best linear fit as shown in Fig. 7. Besides, a calibration stage is also initially carried out.

In view of manufacturer’s recommendation and our initial calibration phase, the output voltage is related to pressure as in Eq. 2:

2 $$ \begin{array}{@{}rcl@{}} V = 0.0278\ast \textit{Pressure}+0.75 \end{array} $$

From Eq. 2, equivalently, the pressure parameter is derived as:

3 $$ \begin{array}{@{}rcl@{}} \textit{Pressure} = (V-0.75)/0.0278 \end{array} $$

#### Bluetooth sensor

**Fig. 8:**
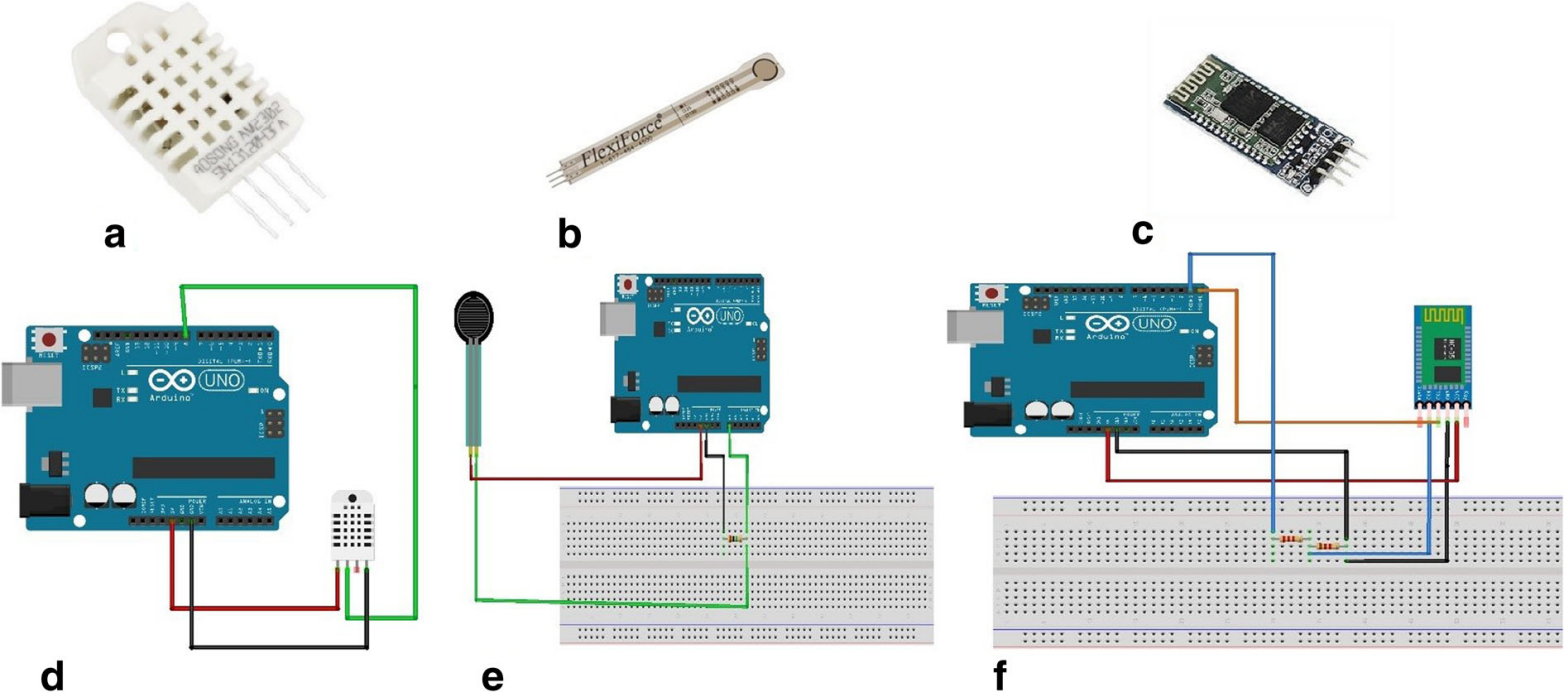
**a** Temperature and humidity. **b** Flexi FSR sensor. **c** HC-05 Bluetooth module. **d** Temperature and humidity schematic design. **e** FSR schematic design. **f** HC-05 schematic design

In order to communicate the sensor outputs (pressure, temperature, and humidity), a communication channel is required. For this purpose, a Bluetooth sensor, which allows us to transfer data over a short distance, up to around 10 m, is employed. We used the HC-05 Bluetooth sensor (see Fig. 8c) because of its simplicity. The module has 6 pins and can easily be interfaced with Arduino board. The logic voltage level of data pin of HC-05 is 3.3 V. Therefore, the connection of data line between Arduino TX and RX needs to connect through a voltage divider in order to not burn the module. On the other hand, the pin of Bluetooth can be connected directly to the Arduino board.

### Android mobile application

**Fig. 9:**
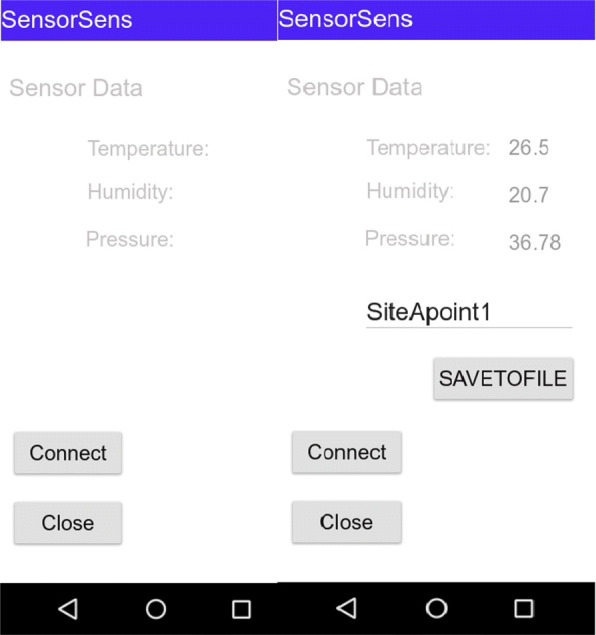
Screenshots of the mobile application

An Android mobile application is developed using Android Studio, which is an open-source software for developing the mobile application and while providing handful support for Android operating system. The purpose for implementing the mobile application was to record the reading from the developed multi-sensor platform. The application uses the Bluetooth communication for acquiring the real-time sensor data from the HC-05 Bluetooth module. The data is then recorded and saved into a csv text file: a screenshot of the running application is highlighted in Fig. 9.

### Data acquisition

**Fig. 10:**
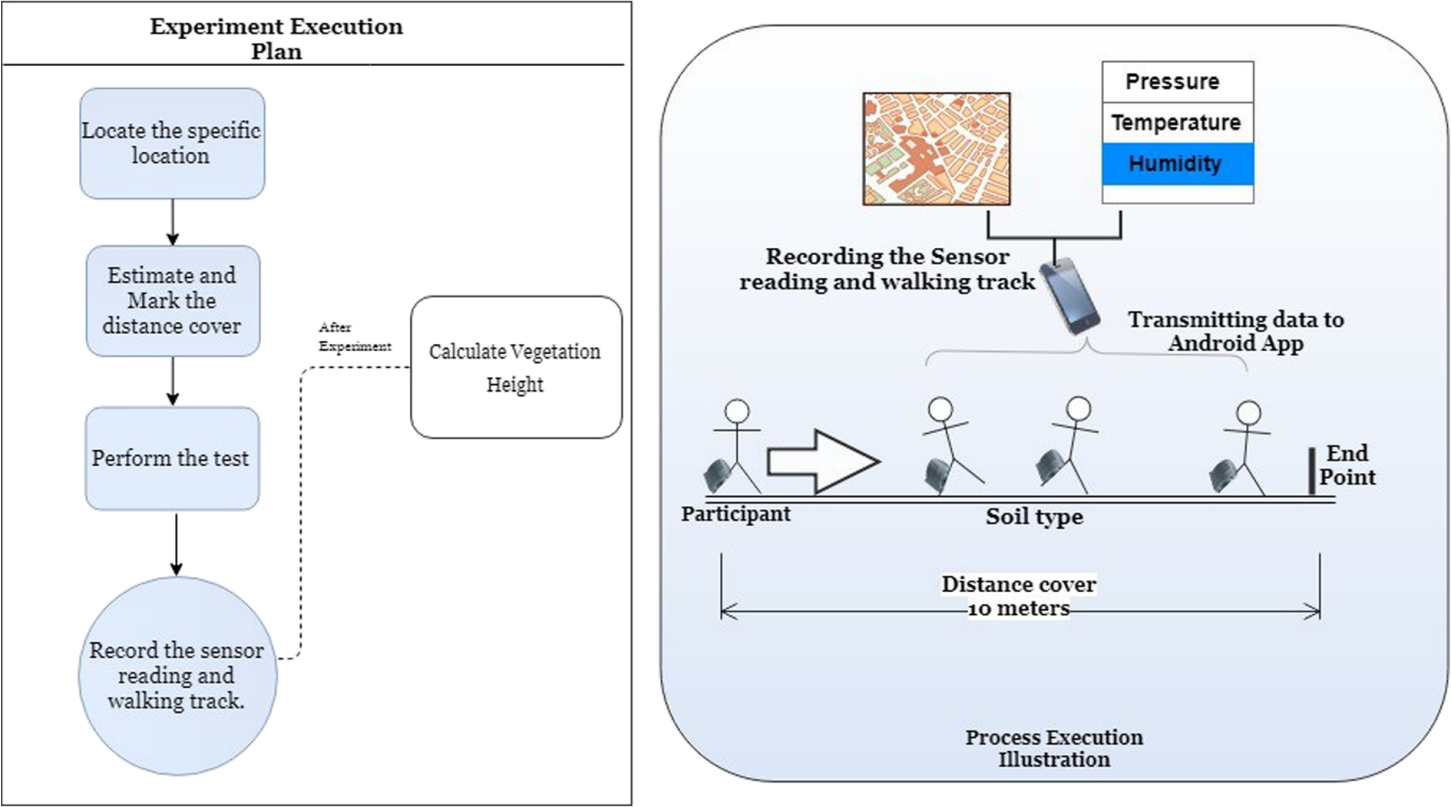
General execution plan of the experiment setup

The acquisition of sensor data is performed during the experimentation stage where around 10 m of distance is covered by a walk at each designated site. During this walk, the wearable platform is attached to the user’s foot. In total, eight tests were performed at each study area where at every test, the path is changed for finding the variation in the sensor data. The general execution plan is shown in Fig. 10, which provides fine experimental details.

### Field measurements and height estimation

**Fig. 11:**
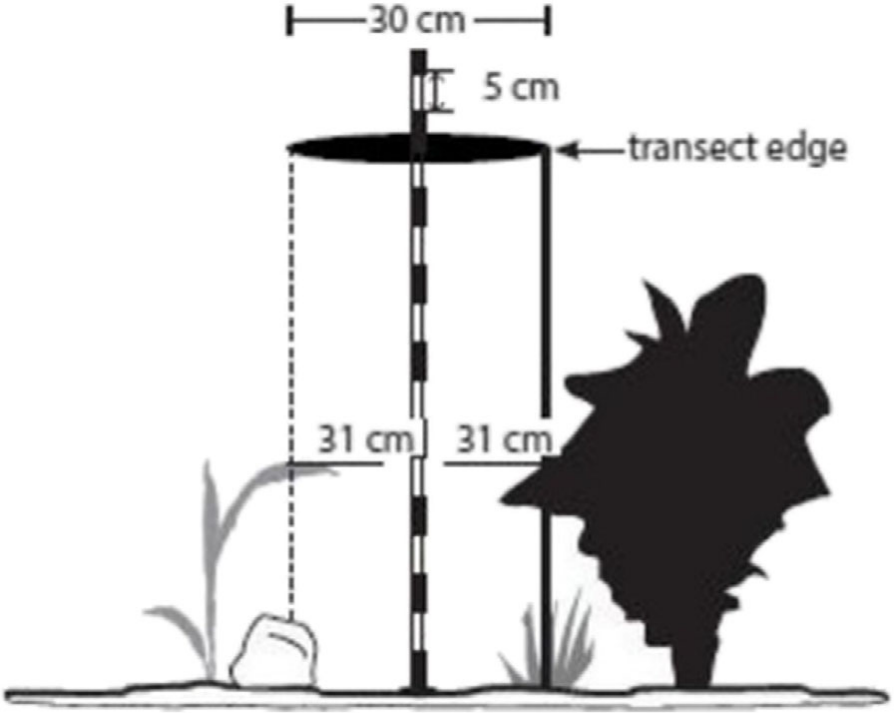
Vegetation field measurement using transcend

In our study, we utilize the line-point intercept method, a popular practitioner-based approach for vegetation height measurement, proposed by Herrick et al. (2014), with some alteration. In this method, the cover is measured along a linear transect line counting the number of “hits” on a target species out of the total number of points measured along that line. In our case, the vegetation height is measured as the height of the tallest plant part within a 30-cm-diameter cylinder projected tangent to transect (Nasim et al. 2019), measured vertically from the soil surface at the center of the cylinder (see Fig. 11), for illustration purpose. Besides, in order to take into account the inherent geometrical constraint of our study area and the density of the plants, we performed the transcend-based measurement five times at regular interval in the region of the study where the plant density is deemed important, and then averaged over. More formally, let H_*i*_ be the *i* th transcend-based vegetation height measurement, so that five distinct measurements H_*i*_ are carried out at each interval of 2.5 m. We then estimate the average maximum vegetation height of canopy cover, over the five measurements (Herrick et al. 2014):

4 $$ \begin{array}{@{}rcl@{}} Avg. VH = \frac15\sum\limits_{i =1,5}{\left( H_{i}\right)} \end{array} $$

The preceding is motivated by the reasonable observation that each of the study fields is associated with at least three distinct vegetation heights (obtained by averaging of transcend measurements according to Eq. 4 in each vegetation type/structure). Indeed, the plant type (either grassland or forest) and structure type (A, B, or C) in each site of study were almost homogeneous in terms of height. Consequently, it genuinely makes sense to consider the vegetation of the same structure, say, A, B, or C to be of the same height. This entails the following. First, the average operation (4) is carried out for each of these structure types present in the study site. Second, the fine-grained variation of the vegetation height at a given structure type is not the prime concern of the study as we hypothesize that it is possible to recognize the structure type using vegetation height information. Third, there exists a mechanism which maps the location to the structure type at each site in order to build the ground truth model, required for the subsequent analysis, which is usually provided through the availability of GPS and Google Maps information. Table 3 exhibits the overall structure of the ground truth dataset where rectangular geometrical approximations were used to model the region in the same site of the same structure type to imitate the bounding box-based reasoning. The developed approach allows us to construct the ground truth in terms of vegetation height for each of the eight study sites.

**Table 3:** Ground truth structure of each study area

Attribute	Description
Vegetation type	Grassland or forest
Structure type	A, B, or C structure
Bounding box	Latitude and longitude of the top left
	and bottom right of the approximate
	rectangular region
Vegetation height	Average vegetation height measured
	using transcend method

### Dataset generation and description

Armed with the developed footwear platform, the user performs normal walking task at each site ensuring that all structure types present in the site are covered. At each walk step, the sensory information is transmitted to the mobile station, and, thereby, to the cloud platform to enable further pre-processing. We particularly focused on pressure sensor output as both the temperature and humidity sensors exhibit no variation due to the experimental setup where all measurements were taken in very short time interval (almost instantaneous) so that the variation of temperature or humidity data is void both within the same site and across different sites. More specifically, after a series of walks at each site using the footwear platform, pressure data are acquired, and their statistics in terms of average pressure value, minimum value, and maximum value are reported. These three entities (average, min, and max of pressure values), whose description is listed in Table 4, are taken as independent variables in our study to infer vegetation height.

**Table 4:** List of independent variables and targeted variable

Independent variables			Method of acquisition	Targeted variable	Methods of calculus
Max pressure	Min pressure	Mean pressure	Sen. platform	Veget. height	Point intercept method

In total, 62 samples or observations are collected during the experiments where 8 experiments were conducted in site 1 (soil type: sand); 8 experiments in site 2 soil type “sand” where all the three vegetation structures A, B, and C were present; 5 experiments in site 3; 8 experiments in site 4; 7 experiments in site 5; 7 experiments in site 6; 8 experiments in site 7; and 8 in site 8. The dataset is split into training and testing datasets, with proportion 80% and 20%, respectively. This was used for training and testing linear regression models.

### Vegetation height estimation using wearable sensors

**Fig. 12:**
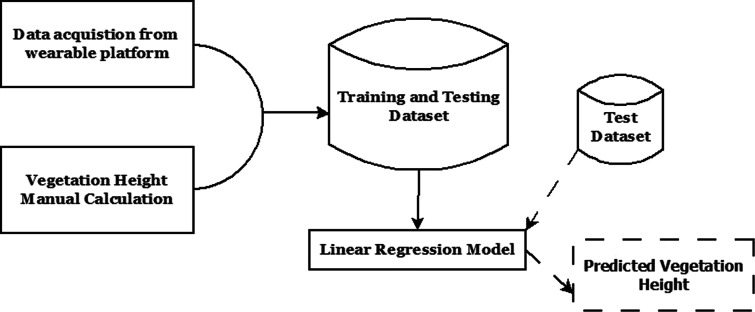
Method of estimation VH

For the purpose of estimating the vegetation height from the pressure measurement, a multi-regression–based approach is devised in order to assess the relevance of the underlined independent variables in this estimation process where the vegetation heights estimated in the field measurement through transcend method are used to determine the parameters of the regression model as highlighted in Fig. 12. Table 5 highlights the pressure attributes employed in the subsequent study.

**Table 5:** Summary of attribute variables of the regression model

Attribute variables	Description
x_1_ = MaxPres	Maximum pressure
x_2_ = MinPres	Minimum pressure
x_3_ = MeanPres	Mean pressure

On the other hand, for the purpose of simplicity and fair results obtained elsewhere, this paper advocates a multi-linear regression model. More specifically, the regression model boils down, for a response variable *y*, to the following:

5 $$ \begin{array}{@{}rcl@{}} y =\beta_{0}+\beta_{1}x_{1}+\beta_{2}x_{2}+\beta_{3}x_{3} \end{array} $$

where *β*_*i*_ (*i* = 0 to 3) are the parameters, to be determined using the training dataset, of the model, interpreted as regression coefficients.

On the other hand, instead of carrying out the regression analysis across all attributes, we have also considered the effects of narrowing down the scope and seeking whether a single attribute will be enough to ensure a good performance in terms of estimating the vegetation height. In other words, this boils down to the following question: *To which extent can a single attributex*_*i*_ (*i* = 1, 3) *estimate the vegetation height?* Statistically speaking, this is equivalent to estimating the extent to which a single linear regression model of *x*_*i*_ is a good fit to estimate the vegetation height of the training dataset. This corresponds to the following fitting equation, where x_*i*_ stands for x_1_, x_2_, or x_3_.

6 $$ \begin{array}{@{}rcl@{}} y =\beta_{0}+\beta_{1}x_{i} \end{array} $$

**Fig. 13:**
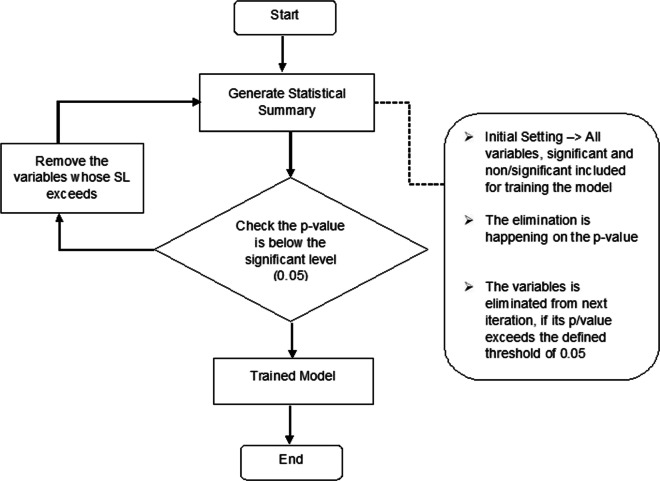
Flowchart of backward elimination technique

This corresponds to a backward elimination–based strategy where we restrict to the most significant attributes as testified by the simple regression fitting outcome Efroymson (1960) with a predefined threshold-based pruning as shown in Fig. 13, instead of treating the three attributes simultaneously, leading to a multi-regression model of three parameters. We set *β*_0_ to 1 for scaling purpose. This yields *β*_1_, *β*_2_, and *β*_3_ to be estimated using the (multi-)regression model(s). The next section details the result of this investigation.

## Results and discussion

### Soil pressure and vegetation height

Intuitively, pressure-based sensors may provide information on land cover such as soil properties, water content, and vegetation properties (density, height, etc.) where the relationship between soil and vegetation is not fully unknown. Indeed, soil compactness, texture, bulk density, and organic/mineral composition directly influence plant growth, quality, and abundance. For instance, Gale et al. (1991) used soil productivity index to predict spruce growth. Landhaeuser et al. (1996) studied the effects of soil compactness on the depth and lateral spread of marsh reed grass. Silva et al. (2008) found that animal trampling can cause soil compactness and degradation of soil structure which negatively affect vegetation growth and height. Similarly, Botta et al. (2006) reinforced Silva et al.’s findings and showed that even an increased frequency of pedestrian or wheel passages can lead to an increase of dry bulk density, which in turn, affects vegetation height. The question can therefore be raised to investigate the extent to which soil patterns can be employed to estimate vegetation height. Especially, is it possible to perform such estimation using solely low-cost sensor platforms? With the recent advances in sensor technologies, including IoT framework, cloud computing, and wearable technology, several breakthroughs in low-cost and efficient environment monitoring technology become accessible to a wider audience (non-specialist group). Indeed, one notices, for instance, a range of wireless sensor networks deployed for habitat and environment monitoring applications; see, e.g., the review paper (Ruiz-Garcia et al. 2009) on the use of smart and low-cost sensors in agriculture, food, and related applications. Zheng et al. (2018) used wireless sensor network (WSN) to conduct the ground-level measurement of snow depth, for understanding the canopy effect. O’shaughnessy et al. (2012) employed WSN along with moving sprinkling system for continuous monitoring of crop canopy temperature. Zhou et al. (2017) put forward a scalable field cost-effective IoT-powered phenotyping platform, referred CropQuant, for crop monitoring and trait measurement in a way to predict vegetation growth. Nevertheless, in the aforementioned studies, one still requires an initial stage of setting up and installing the underlying network and/or sensor infrastructure, which call for cheap alternative solutions.

### Correlation analysis and general trend

**Fig. 14:**
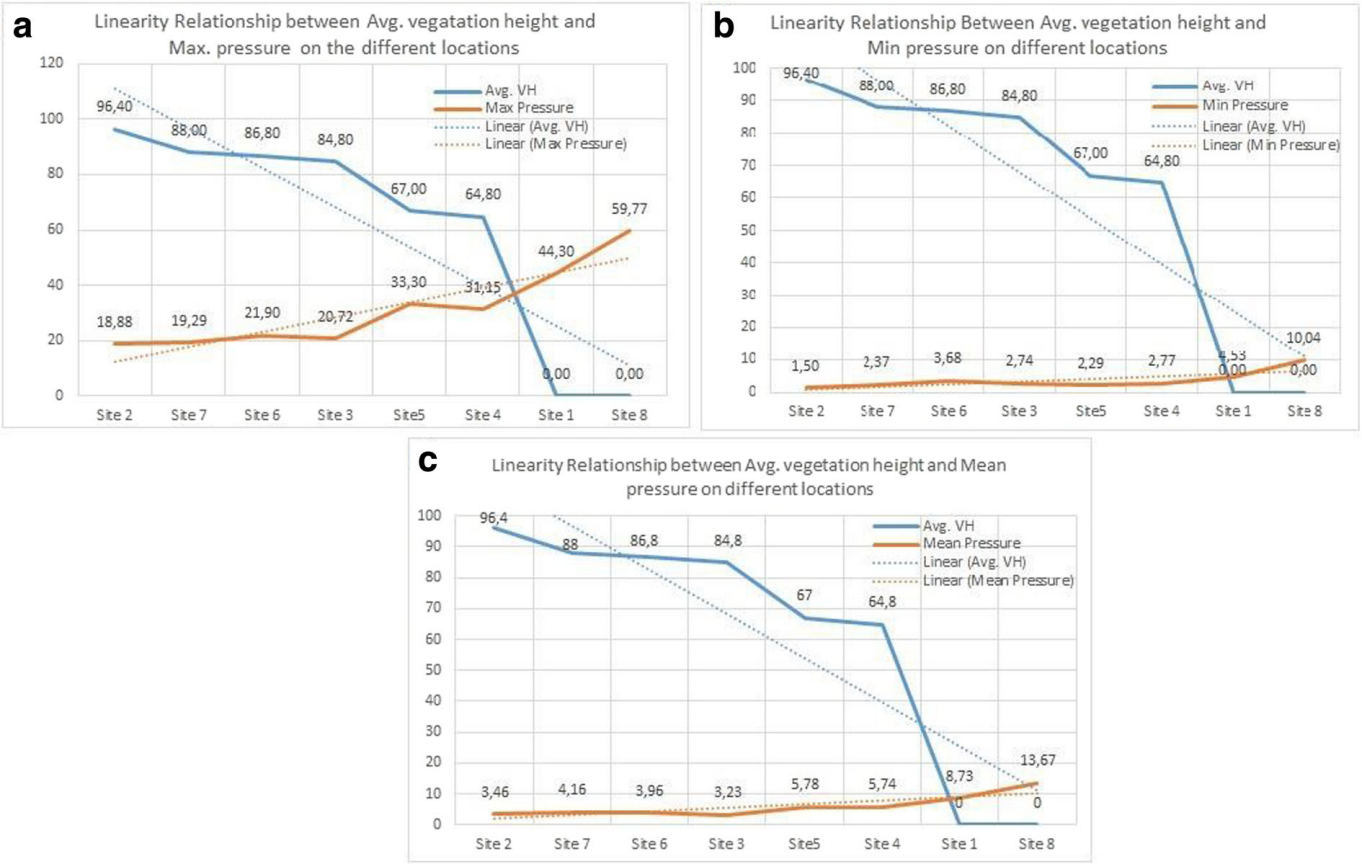
Linearity relation between **a** average maximum pressure (N) and vegetation height (cm); **b** average maximum pressure (N) vegetation height (cm); **c** average maximum pressure (N) and vegetation height (cm)

In order to show the effect of each individual attribute (max-pressure, min-pressure, and mean-pressure) on field measurement of vegetation height as computed using the average expression in Eq. 4, the variation of field-measured vegetation height with respect to individual site is plotted alongside each of the pressure attributes in Fig. 14. On the same plot, we also draw the linear approximation for both field-measured vegetation height and pressure attribute. One notices that any increase (decrease) of the vegetation height is translated into either an increase or a decrease of the pressure attribute value, except for site location A (sand), where both vegetation height and pressure values are meaningless. On other hand, the direction of variation (either increase or decrease) with respect to that of vegetation height indicates a positive or a negative correlation of the given attribute with respect to vegetation height. In this respect, Fig. 14a highlights a rough negative correlation of maximum pressure with vegetation height (a slight deviation can be observed in sites F, C, E, and D but without changing the overall trend). To confirm this trend, the calculus of the Pearson correlation coefficient between the attribute variable and the vegetation height indicates a correlation value of *r* = − 0.9451 with *p* value 0.0013, which testifies of high statistical significance at 5% significance level. Similarly, Fig. 14c indicates that the mean pressure negatively correlates with the vegetation height as the corresponding Pearson correlation coefficient reads *r* = − 0.9219 and *p* value = 0.0011. However, the min-pressure attribute does not exhibit the same behavior since the corresponding Pearson correlation coefficient reads *r* = − 0.79 but *p* value = 0.02. On the other hand, the linear fit of individual attribute evolution shown in Fig. 14 indicates that the linear interpolation is roughly tenable, especially for mean and min-pressure attributes.

**Fig. 15:**
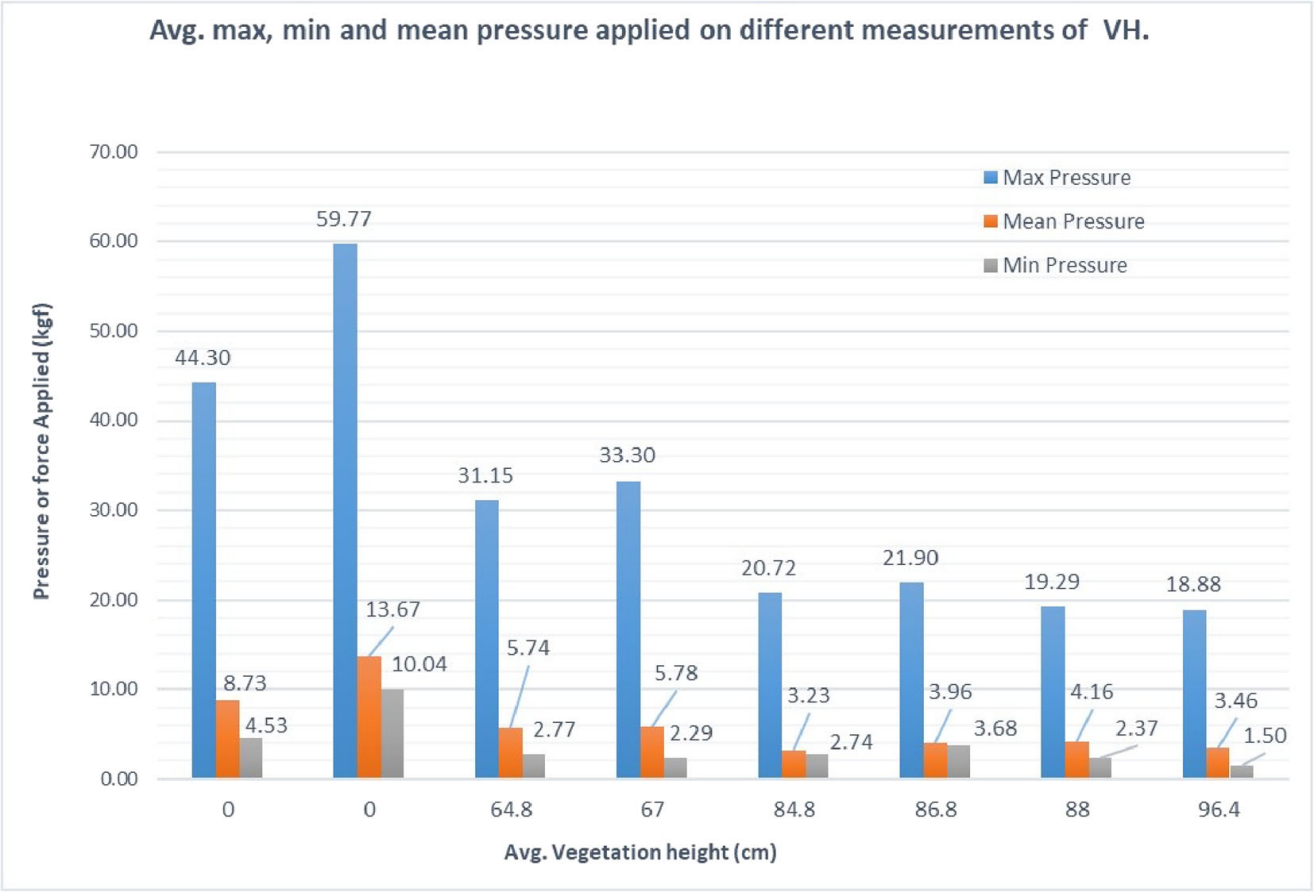
Pressure attributes applied on different heights of vegetation

Moreover, in order to comprehend the order of magnitude of the variations of the attribute variables in different site locations, Fig. 15 exhibits the overall quantification of the three attributes (mean-pressure, max-pressure, and min-pressure) at each site location. The plot indicates that higher pressure readings are observed when exposed to more stiff surface such as asphalt and sand, whereas stiffness may exhibit higher variations in lands where different levels of vegetation are observed. The results also demonstrate that sites with high vegetation height yield lower values of pressure attributes. To explain this observation, one notices that small stiffness around the surface is often due to the presence of irregularities in the vicinity area, which causes walking difficulty that is translated into low pressure values.

For validating and verifying the results, we later applied statistical analysis, in order to find out whether the associated attributes show any significance level of correlation with the targeted variable. This is detailed in the next section.

### Attribute variability and relevance

In order to assess the viability and ability of each of the pressure attributes to discriminate the various vegetation heights, Table 6 summarizes the key statistics related to attributes x_1_, x_2_, and x_3_ over each study site and for each vegetation type. A simple reading of the measurement trend indicates, for instance, that if the maximum pressure attribute is taken alone, we cannot discriminate between grassland A (type B, in site B) and forest C (type A in site F) as they yield very close maximum pressure values. However, when considering the other two attributes (minimum pressure and mean pressure), it becomes intuitively possible to discriminate between the two cases. This exemplification is further generalized later on and reinforced by findings from correlation analysis.

**Table 6:** Metrics derived from pressure sensor reading

Study area	Structure	Vegetation type	Max pressure	Min pressure	Mean pressure	Standard deviation
Site A	Sand	No type	–	–	–	–
		Type A–B	16.85	2.51	2.49	4.49
Site B	Grassland A	Type B–B	19.54	2.35	4.79	6.42
		Type C–B	17.61	3.27	3.6	5.18
Site C	Forest A	Type A–C	13.88	1.01	2.80	4.60
		Type B–C	22.39	4.62	3.86	6.61
		Type C–C	20.61	1.93	3.22	6.28
Site D	Grassland B	Type A–D	30	2.20	4.98	9.51
		Type B–D	37.03	1.93	6.19	10.85
Site E	Forest B	Type A–E	41.83	2.66	8.23	11.67
		Type B–E	38.53	1.77	7.60	10.83
Site F	Forest C	Type F–A	19.54	4.31	3.25	5.59
		Type F–B	21.65	8.20	5.60	8.07
		Type F–C	31.96	10.89	5.72	8.70
Site G	Forest D	Type G–A	22.69	1.31	22.69	6.56
		Type G–B	20	0.43	5.32	6.84
		Type G–C	18.35	5.35	5.43	6.39
Site H	Road	No type	–	–	–	–

More specifically, we first compute Pearson’s correlation to find the significance correlation between each attribute (minimum pressure, maximum pressure, and mean pressure) with the vegetation height, where unlike averaged outcomes over each site shown in Section “Correlation analysis and general trend”, the whole attribute readings are considered in this case. The results summarized in Table 7 indicate that there is a weakly significant inverse relationship between the min pressure and vegetation height *r*(61) = − 0.39, *n* = 62, *p* << 0.00*. In contrary, Table 7 also indicates a strong statistically negative correlation observed between the maximum pressure and vegetation height *r*(61) = − 0.86, *n* = 62, *p* << 0.00*. Similar relationship holds for the mean pressure output and the vegetation height where it was found *r*(61) = − 0.85,*n* = 62,*p* < 0.00 ∗.

**Table 7:** Pearson’s correlation coefficient max, min and mean pressure vs. VH and regression analysis

Variable	Sample			95 % Clevel
	VH value	Correlation	*p* value	Reg. coef.
Max. Pres.	62	− 0.86	< 0.00*	*β* = − 2.11
Min. Pres.	62	− 0.39	< 0.00*	*β* = − 2.81
Mean Pres.	62	− 0.85	< 0.00*	*β* = 8.74

Especially, Table 7 exhibits the regression coefficient when a linear fit between the underlined independent variable (max-pressure, min-pressure, or mean-pressure) and vegetation height is enforced.

Clearly, the small value of Pearson coefficient in Table 8 indicates again the min pressure attribute should be discarded and would not predict the vegetation height appropriately.

**Table 8:** Statistical summary after first iteration of multi-regression model

Regression statistics						
Multiple R	0.880					
*R* square	0.774					
Adjusted *R* square	0.762					
Standard error	18,291					
Observations	61					
ANOVA						
	df		MS	F	Significance F	
Regression	3	65,364.73	21,788.24	65.13	< 0.00	
Residual	57	19,069.88	334.56			
Total	60	84,434.61				
	Coefficients	Standard error	*t* Stat	*p* value	Lower 95%	Upper 95%
Intercept	123,587	5.533	22,337	< 0.000	112,507	134,667
Min-pressure	0.743	0.611	1.216	0.229	− 0.481	1.966
Max-pressure	− 1.067	0.423	− 2.525	0.014	− 1.914	− 0.221
Mean-pressure	− 5.287	1.997	− 2.648	0.010	− 9.286	− 1.289

### Multi-regression results

Results of Sections “Correlation analysis and general trend” and “Attribute variability and relevance” showed that the max and mean-pressure exhibit strong and statistically significant negative correlation with vegetation height when considering either average site values (as in Section “Correlation analysis and general trend”) or the whole readings (as in Section “Attribute variability and relevance”). Initially, we apply the multi-regression model with backward elimination method while all the independent variables (min-, max-, and mean-pressure) are taken into account during the training phase of the model. After we trained the model, we computed the *p* value for each attribute that is then compared to some predefined significance threshold. The latter triggers the decision to maintain or discard the corresponding attribute variable (see Fig. 13). Table 8 highlights the statistical summary obtained after the first iteration. Especially, we have conduced the analysis of variance (ANOVA) to identify the level of variability within the corresponding regression model and quantifies the significance level. In this regard, the table shows that the min-pressure attribute does not yield a statistically significant result (*p* value = 0.229). This is in agreement with the correlation analysis performed in Section “Correlation analysis and general trend”, whereas the maximum and mean attribute *p* values are consistent with 5% significance level. This indicates that the min-pressure attribute is a statistically low significant parameter to be considered as a powerful predicator for the training model. Therefore, considering such an elimination-based analysis, the next iteration is run without the min-pressure attribute. The results of this subsequent analysis are shown in Table 9.

**Table 9:** Statistical summary of final iteration of multi-regression model

Regression statistics						
Multiple *R*	0.877					
*R* square	0.768					
Adjusted *R* square	0.760					
Standard error	18,366					
Observations	61					
ANOVA						
	df		MS	F	Significance F	
Regression	2	64,870.22	32435.11	96.16	< 0.00	
Residual	58	19,564.39	337.32			
Total	60	84,434.61				
	Coefficients	Standard error	t Stat	*p* value	Lower 95%	Upper 95%
Intercept	123,978	5.546	22,353	< 0.000	112,876	135,080
Max	− 1.266	0.392	− 3.233	0.002	− 2.050	− 0.482
Mean	− 3.885	1.637	− 2.374	0.021	− 7.161	− 0.609

On the other hand, two performance metrics were employed to quantify the performance of the multi-regression model; namely, the standard root mean squared error (RMSE) and *R*-squared. The latter indicates the percentage of the variance in the dependent variable that the independent variables explain collectively, measuring the strength of the relationship between the regression model and the dependent variables. These are employed as performance metrics for the evaluation of the multi-regression model and calculus of regression coefficients using the training dataset. The predictive performance of the linear regression and multi-regression model in terms of two evaluation measures for a single target variable is presented in Table 10.

**Table 10:** Evaluating the performance of linear regression and multi-regression for estimating VH

Attributes	*R* squared	RMSE (cm)	Model
Max pressure	0.81	16.73	127.03–2.12*x1
Max and mean pressure	0.83	15.75	122.91–1.25*x1–3.75*x3

## Conclusion

In this study, a data acquisition approach from a locally developed ubiquitous sensor’s wearable platform, for predicting the vegetation height, has been proposed and evaluated. The approach is based on developing a machine learning model to learn the vegetation height from key attributes associated to pressure, temperature, and humidity measurements. The idea consists of exploring the variation of pressure attribute from the wearable measurement platform at different levels of vegetation height. The approach uses a multi-regression model that involves pressure-related attributes (minimum-pressure, maximum-pressure, and mean-pressure). The experimental setup and its time frame discarded any impact of temperature and humidity information on the output, leaving only the pressure indicator as a relevant parameter to investigate further. The correlation and statistical analyses showed that the maximum-pressure and the mean-pressure are more significant in predicting the vegetation height. Thereby, both single and multi-regression models were appropriately designed and tested. In general, the results acquired from the developed approach are not meant to outperform or even approach some state-of-the-art models that use elaborated remote sensing or satellite imaging techniques, but will pave the way for the development of low-cost ubiquitous technology. Indeed, contrary to satellite imaging and advanced remote sensing technology that demand high operational costs, time, and labor, our approach entitles new opportunities toward data acquisition at low cost, and less time and labor demanding. However, this is a pilot approach and much work is still needed to be done in order to construct a more efficient machine learning model that takes into account user’s various modalities and possibly integrating other soil-related sensory information. In addition, our approach provides the feasibility for estimating minimalistic characteristic of forest structure nearly at very low cost and less labor demand. The results encourage future research in data acquisition methods from wearable ubiquitous sensor platforms in vegetation height estimation without the use of active sensors, such as LiDAR, or the need of extensive field campaigns. This can reduce the operational and labor cost and facilitate the ecological monitoring and environmental sustainability planning.
